# The Organised Practice of Massage

**Published:** 1921-04-23

**Authors:** 


### THE ORGANISED PRACTICE OF MASSAGE.

The annual meeting of the Chartered Society of Massage and Medical Gymnastics, held on April 16, at the Armitage Hall, Great Portland Street, promised to be of more than usual interest. The first annual report of the new Chartered Society—the successor to the Incorporated Society of Trained Masseuses—was to be presented by the Chairman of the Council, Sir Cooper Perry, M.D., and the agenda was not confined to the usual formal and dry business of annual meetings, but included the discussion of several vital matters of professional character. Unfortunately, though these matters were ably presented, the audience did not take advantage of the opportunity for discussion, and only in one case—the question of Red Cross Clinics and civilian patients—on which it was particularly desired to ascertain the opinion of members, were any points raised.

### The Society's First Year.

The report of the Council shows great activity in the work of the year. During 1920, 20,294 letters were received and 27,515 sent out. Ten examinations were carried out by the new Society and 1,426 certificates granted. Several training-schools have been able to extend their training to medical gymnastics and medical electricity, and the Council looks forward to the establishment of conjoint training at all schools in the near future. Assistance has been given to various hospitals and clinics in the formation of classes on a uniform basis. The demand for the formation of Lecture Centres is increasing, but the complete satisfaction of this demand depends on an improved financial outlook; members are reminded that, except on special occasions, lectures are free to members and open to certificate-holders and students in training for the Society's examination at the small fee of 1s. a lecture, lecturers' fees and the incidental expenses of the Centres being met from the Society's general funds. Of the 4,200 members of the Incorporated Society of Trained Masseuses, only 1,822 have as yet become members of the Chartered Society, a matter of disappointment to the Council; of the total number certificated from the Incorporated Society—6,735 men and women—2,148 have registered with the Chartered Society. It is hoped that the remainder will do so by July 19, in time for their names to appear in the first printed Register of qualified workers to be published in the autumn, which will give the names and addresses of all registered masseuses and masseurs. In presenting the balance-sheet, Sir Cooper Perry drew attention to the appreciation in the market price of the Society's investments, and pointed out the necessity to increase investments by at least another £12,000, as against a capital of £6,796 shown in the balance-sheet for 1920. This can be produced if the 6,000 odd certificate holders of the Incorporated Society will register with the Chartered Society.

For the election of Council members 2,400 voting papers were sent out, and 928 valid and 35 invalid papers were returned. Miss Angove and Miss Field were elected as teachers in recognised schools, and the Hon. Essex French and Miss E. Winifred Bliss as non-teachers.

### A Lack of Imagination.

Mr. Louis H. M. Dick spoke to the meeting on the value of the Members' Fund, a small benevolent fund inaugurated by the Incorporated Society, which is available for registered members of the Chartered Society and for the members of the Incorporated Society at the time of its reconstitution. If only two-thirds of 6,000 members will each give the small sum of 1s. a year—a farthing a week—the result will be an income of £200 a year for the fund. He then discussed the general question of sickness and accident insurance, pointing out that it requires imagination to realise the immense value of insurance, and that though women do not like insurance because they do not always get immediate benefit for what they are paying for, nurses, from the knowledge gained in their calling, should be the last people to refrain from this necessary precaution. He is confident that knowledge of the number and circumstances of one day's claims on the Royal National Pension Fund would cause every nurse immediately to take out an insurance. He touched on the conditions of the accident and illness insurance policy arranged by the Society with the Eagle, Star, and British Dominions Insurance Company, which pays to the Council an agency fee on all premiums received from registered members of the Chartered Society. These fees, after the payment of expenses, are used for the benefit of insured members through the Members' Fund.

Red Cross Clinics and Civilian Patients.

Miss Grafton, following up her letter in the March *Journal*, asked for the views of members on this question, the point being, Would the carrying on of these clinics for the treatment of civilian cases be likely to be economically harmful or good so far as the individual practising masseuse is concerned? The Misses Noyes, Long, Oswald, Lucas, Murdoch, Kirby, and others raised certain points, but sufficient consideration did not seem to have been given to the subject to elicit any considered opinion. The Chairman promised consideration by the Council to the various points, and that an endeavour will be made to ascertain what policy the British Red Cross Society intends to pursue in regard to the administration and management of these clinics.

The last matter for discussion was "A proposed Club for professional women to include members of the Chartered Society." Sir Cooper Perry pointed out that the Cowdray Club given to the College of Nursing, which is now being proceeded with, is to be open to professional women other than College members and trained nurses, it having been considered that a wider basis of membership than limitation to nurses would be of more advantage to nurses and all concerned, a statement which was received with applause. He has enlisted Lady Cowdray's sympathy and interest in the profession of massage, and she will be glad to welcome registered members of the Chartered Society as members of the Cowdray Club, which will be managed by a Club Committee, which must in the first place be nominated, but ultimately will be elected. He hopes the Club will be running in twelve months' time.

After thanks to the National Institute for the Blind for the use of the hall, and to Sir Cooper Perry for presiding, the meeting broke up, and those present adjourned to Mortimer Hall to enjoy the hospitality and excellent tea provided by the Council of the Chartered Society.

